# Single-Cell RNA-seq Identification of the Cellular Molecular Characteristics of Sporadic Bilateral Clear Cell Renal Cell Carcinoma

**DOI:** 10.3389/fonc.2021.659251

**Published:** 2021-06-08

**Authors:** Zhenyuan Yu, Wenhao Lu, Cheng Su, Yufang Lv, Yu Ye, Bingqian Guo, Deyun Liu, Haibiao Yan, Hua Mi, Tianyu Li, Qingyun Zhang, Jiwen Cheng, Zengnan Mo

**Affiliations:** ^1^ Department of Urology, The First Affiliated Hospital of Guangxi Medical University, Nanning, China; ^2^ Center for Genomic and Personalized Medicine, Guangxi Medical University, Nanning, China; ^3^ Institute of Urology and Nephrology, The First Affiliated Hospital of Guangxi Medical University, Nanning, China; ^4^ Guangxi Collaborative Innovation Center for Genomic and Personalized Medicine, Guangxi Medical University, Nanning, China; ^5^ Guangxi Key Laboratory for Genomic and Personalized Medicine, Guangxi Key Laboratory of Colleges and Universities, Nanning, China; ^6^ Scientific Research Department, The Second Affiliated Hospital of Guangxi Medical University, Nanning, China; ^7^ Department of Urology, Affiliated Tumour Hospital of Guangxi Medical University, Nanning, China

**Keywords:** single-cell RNA sequencing, sporadic bilateral clear cell renal cell carcinoma, tumour cellular molecular characteristics, single-cell RNA atlas, tumour cell microenvironment

## Abstract

Bilateral renal cell carcinoma (RCC) is a rare disease that can be classified as either familial or sporadic. Studying the cellular molecular characteristics of sporadic bilateral RCC is important to provide guidance for clinical treatment. Cellular molecular characteristics can be expressed at the RNA level, especially at the single-cell degree. Single-cell RNA sequencing (scRNA-seq) was performed on bilateral clear cell RCC (ccRCC). A total of 3,575 and 3,568 high-quality single-cell transcriptome data were captured from the left and right tumour tissues, respectively. Gene characteristics were identified by comparing left and right tumours at the scRNA level. The complex cellular environment of bilateral ccRCC was presented by using scRNA-seq. Single-cell transcriptomic analysis revealed high similarity in gene expression among most of the cell types of bilateral RCCs but significant differences in gene expression among different site tumour cells. Additionally, the potential biological function of different tumour cell types was determined by gene ontology (GO) analysis. The transcriptome characteristics of tumour tissues in different locations at the single-cell transcriptome level were revealed through the scRNA-seq of bilateral sporadic ccRCC. This work provides new insights into the diagnosis and treatment of bilateral RCC.

## Introduction

Renal cell carcinoma (RCC) is a common tumour in the urinary system with lower incidence rate compared with bladder and prostate cancers (1). As a malignant tumour, RCC is derived from renal tubular epithelial cells (2). Most RCC cases have a better prognosis than other cancers in the urinary system, such as upper urinary tract urothelial carcinoma and muscular infiltrating bladder carcinoma (1, 3). However, this rate has been increasing in the past decades (4, 5). RCC can be divided into several histopathological subtypes, such as clear-cell RCC (ccRCC), papillary RCC (pRCC), chromophobe RCC (chRCC) and other rare subtypes, which accounted for 80%, 10%–15% and 4%–5% of RCC, respectively (6). Bilateral RCC is a rare type of RCC that accounts for 1% to 5% RCC cases (7, 8) and can be categorised as familial and sporadic. Although the incidence rates of bilateral RCC are not high, studying this disease will help understand its progress and prognosis.

In addition to familial aggregation, germline alterations in DNA level occur in most cases of familial bilateral RCC. For example, VHL disease is caused by a germline mutation in *VHL* gene and always leads to bilateral RCC (9). Research on sporadic bilateral RCC usually falls into the clinical retrospective category (10, 11). Only a few works have revealed the molecular characteristics of bilateral RCC at the cellular level. Exploring the molecular characteristics of tumours at the cellular level may help further understand the complexity of bilateral RCC.

With further development, high-throughput single-cell RNA sequencing (scRNA-seq) (12) has been applied in many urinary organs, such as bladders, prostates and kidneys (13–15). In addition, scRNA-seq has been widely used in the study of various tumours. For example, the intra-tumoural heterogeneity and malignant progression in pancreatic ductal adenocarcinoma have been revealed by scRNA-seq (16). And by using this new technique, the developmental lineage of tumour cells, the tumour microenvironment and potential therapeutic targets can be revealed, such as in oligodendroglioma, breast cancer, prostate cancer and hepatocellular carcinoma (17–20). In this study, a case of sporadic bilateral clear cell RCC (ccRCC) was examined at single-cell resolution by scRNA-seq. The single-cell transcriptomic characteristics of bilateral tumour tissues were determined with this new technique.

## Materials and Methods

### Bilateral ccRCC Sample

**Figure 1:**
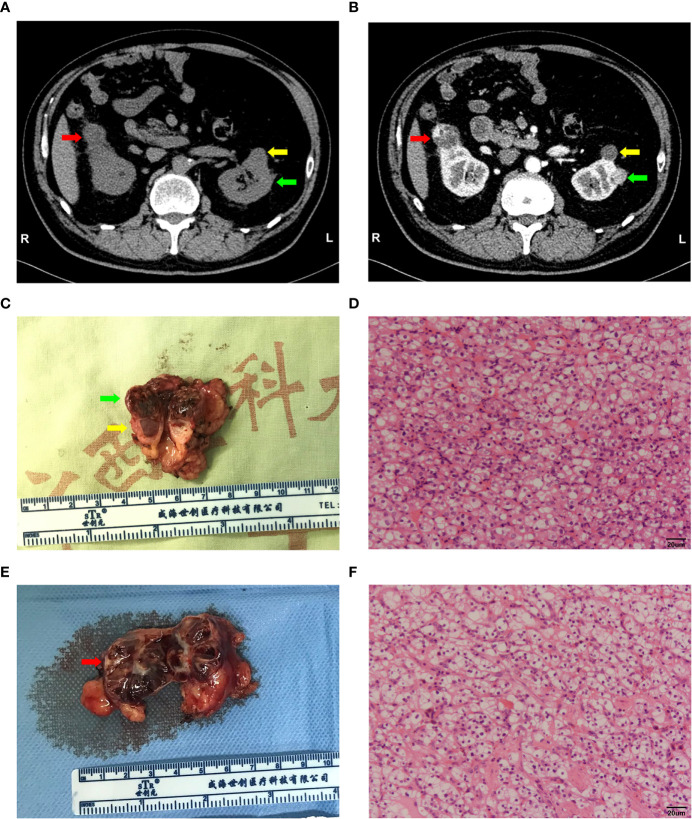
Imaging and pathological information of this sample. **(A, B)** Computed tomography (CT) plain and arterial images of the bilateral tumour. Right tumour (red arrow), left tumour (green arrow) and renal cyst (yellow arrow). **(C, D)** General view of the left renal tumour and histological morphology of the cells by HE staining. Scale bars, 20 μm. **(E, F)** General view of the right renal tumour and histological morphology of the cells by HE staining. Scale bars, 20 μm.

The sample was obtained from a patient (**Table 1**) undergoing robot-assisted laparoscopic bilateral partial nephrectomy in the first affiliated hospital Guangxi Medical University in China. Preoperative CT examination of the patient indicated bilateral renal tumours and left renal cyst (**Figure 1A**). The tumours showed signs of enhancement in the arterial phase (**Figure 1B**). Postoperative pathological results revealed that both tumours were ccRCC (right tumour under WHO/ISUP grade I and left tumour under WHO/ISUP grade II, **Figures 1C–F**). The patient’s family had no history of similar diseases. Neurological, fundus, and pancreatic lesions were not observed in the patient. Thus, the diagnosis was sporadic bilateral ccRCC.

**Table 1:** The information of patient.

Sex	Age	Family	Fundus lesions	Pancreatic lesion	Histology
Male	64	No	No	No	ccRCC

### Isolation of Single-Cell Suspension

Fresh bilateral tumour samples were obtained from the hospital and transferred to the laboratory in cold transfer buffer (HBSS, Gibco, C11875500BT; 5% FBS, Gibco, 10099141 and 1% penicillin/streptomycin, Gibco,15240062) within 30 min.

After being washed with 4°C DPBS (WISENT, 311-425-CL), the tissue samples were cut into 2- to 4-mm pieces with sterile scissors, washed again and resuspended in pre-cold DPBS twice. The tissue specimens were digested for 30 min at 37°C in a digestion solution containing 1 mg/mL collagenase I (Gibco, 5401020001) and 1 mg/mL DNaseI (Roche, 10104159001) in HBSS, and the supernatant was subsequently removed. The whole digestion process required a slight jolt and was terminated by adding 10 mL of DMEM (WISENT, 319-006-CL) with 10% FBS (Gibco, 10099141) and using a 70-μm cell strainer (Falcon) to filter out large tissue fragments. The cells were washed twice with pre-cold DPBS with 1% FBS and centrifuged at 300 g for 5 min. Red blood cells (RBCs) were removed using 5 mL of RBC lysis buffer (10× diluted to 1×; BioLegend, 420301) for 5 min on ice and then filtered through a 40 μm cell strainer. The cells were washed twice with DPBS and resuspended in appropriate volume DPBS with 1% FBS to obtain the single-cell suspension. Viable cells were counted after trypan blue (Gibco, 15250-061) staining (**Table 2**).

**Table 2:** Information and sequencing statistics of bilateral renal cell carcinoma samples.

Sample ID	Tumor location	WHO/ISUP	Cell viability (%)	Number of cells	Median number of detected genes	Sequencing saturation (%)	Mean reads per cell	Number of cells post filtering
ccRCC1	Right	Grade I	86.1	6,348	1,794	65.0	49,794	3,568
ccRCC2	Left	Grade II	85.2	7,775	1,160	48.9	31,270	3,575

### Single-Cell cDNA Library Construction and Sequencing

ScRNA-seq was performed on the above single-cell suspensions in accordance with the standard protocol in the user guide of 10× Genomics Chromium Single Cell 3′ Reagent Kit V3 (https://support.10xgenomics.com/single-cell-gene-expression/index/doc/user-guide-chromium-single-cell-3-reagent-kits-user-guide-v3-chemistry). The concentration of the single-cell suspensions was manually counted using a haemocytometer and adjusted to 2000 cells/μl. Appropriate volume of 10,000 cells was calculated in each sample. The samples were then loaded into a 10× genomics single-cell chip. After droplet generation, the samples were transferred onto a PCR tube, and reverse transcription reaction was performed using T100 Thermal Cycler (Bio-Rad). cDNA was recovered using a recovery agent provided by 10× Genomics, followed by silane DynaBead clean up as outlined in the Kit V3 user guide. Prior to clean-up using SPRIselect beads, the cDNA was amplified for 11 to 12 cycles.

All samples were sequenced using Hiseq Xten (Illumina, San Diego, CA) with the following run parameters: read 1 for 150 cycles, read 2 for 150 cycles, and index for 14 cycles. Preliminary sequencing files (.bcl) were converted to FASTQ files on CellRanger (version 3.0.2, https://support.10xgenomics.com/single-cell-gene-expression/software/pipelines/latest/what-is-cell-ranger). FASTQ files were compared with the human genome reference sequence GRCh38. With the use of CellRanger, a barcode table, a feature table and a gene expression matrix were generated.

### Seurat for Quality Control (QC) and Secondary Analysis

**Figure 2:**
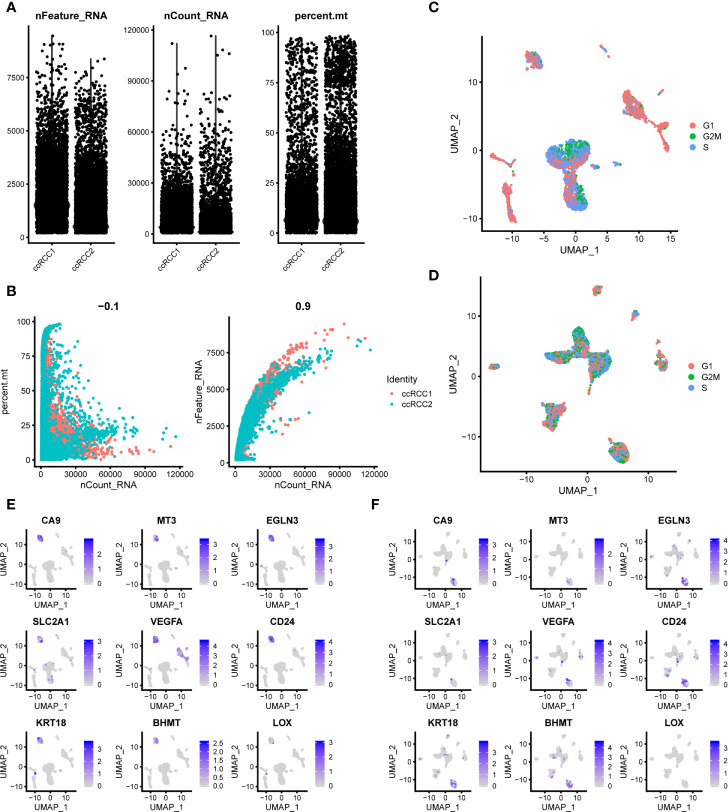
Quality control (QC) of the scRNA-seq data. **(A)** QC of bilateral ccRCC scRNA-seq data. nFeature, number of genes; nCount, unique molecular identifiers (UMIs); percent.mt, percentage of mitochondrial genes. **(B)** Relationship between the percentage of mitochondrial genes and the mRNA reads and between the amount and reads of mRNA. **(C, D)** Cell clustering results by eliminating the influence of cell cycle genes. **(E, F)** The DEGs of ccRCC cells reported in previous study also verified in ccRCC1 **(E)** and ccRCC2 **(F)**.

R (version 3.5.2, https://www.r-project.org/) and Seurat (21, 22) R package (version 3.1.1, https://satijalab.org/seurat/) were used for QC and data secondary analysis, respectively. Generally, the number of genes more than 200 but less than twice of the median number of detected genes (potential cell duplets) was considered as a cell. In accordance with the median number of genes, the percentage of mitochondrial genes and the relationship between the percentage of mitochondrial genes and mRNA reads (**Figures 2A, B**), filtering was conducted on sample ccRCC1 cells (the median number of detected genes was 1794) with < 200 and > 3500 genes and a mitochondrial gene percentage of > 15% and on sample ccRCC2 cells (the median number of detected genes was 1,160) with < 200 and > 2500 genes and a mitochondrial gene percentage of > 15%. High-quality ccRCC cells were then obtained, and the numbers of ccRCC1 and ccRCC2 were counted as 3568 and 3575, respectively (**Table 2**).

When single-cell data normalisation was completed, the highly variable genes were identified after controlling for the relationship between average expression and dispersion. The top 2000 genes were used for downstream analysis. Cell cycle analysis was performed on ccRCC1 and ccRCC2 to eliminate the effect of cell cycle genes (**Figures 2C, D**). Significant principal components (PCs) were identified by the *Jackstraw* function. Among the PCs labelled as the input for uniform manifold approximation and projection (UMAP), 20 were statistically significant. With a resolution of 0.5, all the cells were clustered by *FindClusters* function. *FindAllMarkers* function was subsequently applied to calculate differentially expressed genes (DEGs) between each type of cell (**Tables S1**, **S2**).

### Copy Number Variation (CNV) Analysis of Single-Cell Data

InferCNV (https://github.com/broadinstitute/inferCNV) was used to perform CNV analysis on scRNA-seq data. InferCNV is a tool which can use single-cell RNA-Seq expression to visualize CNV in cells. Initial CNVs were estimated by analysing the gene including their chromosomal locations and averaging their relative expression values (23, 24). Cell types were initially classified by using the Seurat package. InferCNV was then applied to calculate the CNV on all euchromosomes for each cell type. For 10× Genomics single-cell data, the cut-off value was 0.1.

### Comparison of Different ccRCC Samples

The overall similarity of gene expression between two samples was calculated by determining the average expression amount of each gene in the two samples. Pearson correlation coefficient for the average expression amount of each gene was also computed for similarity comparison. The average gene expression was transformed by the formula *log1p*. Pearson’s correlation coefficient for the similar cell type between two different samples was also calculated with the same method.

### Gene Ontology (GO) Enrichment Analysis on Tumour Cells

The top 50 DEGs (**Tables S1**, **S2**) between ccRCC1 and ccRCC2 types were selected for GO enrichment analysis (25) (http://geneontology.org/). Each tumour cell type underwent enrichment analysis for biological process, and the 15 most significant biological processes were selected. Two tumour cells (ccRCC2) were unable to undergo enrichment on significant biological processes.

### Comparing Present scRNA-seq Data With Those of Normal Kidney and ccRCC

The scRNA-seq data of normal kidneys came from our previous study, GSE131685 (15). The scRNA-seq data of ccRCC were obtained from a previous study (26). From previous study, we screened out nine genes (*CA9, MT3, EGLN3, SLC2A1, VEGFA, CD24, KRT18, BHMT* and *LOX*) that specifically expressed in ccRCC cells. We found that all of these genes were specifically highly expressed in ccRCC1 tumour cells (**Figure 2E**). And all the genes except *LOX* were specifically highly expressed in ccRCC2 tumour cells (**Figure 2F**). The reliability of our data is further verified by comparison.

## Results

### scRNA-seq Revealed the Complex Cellular Environment of Bilateral ccRCC

**Figure 3:**
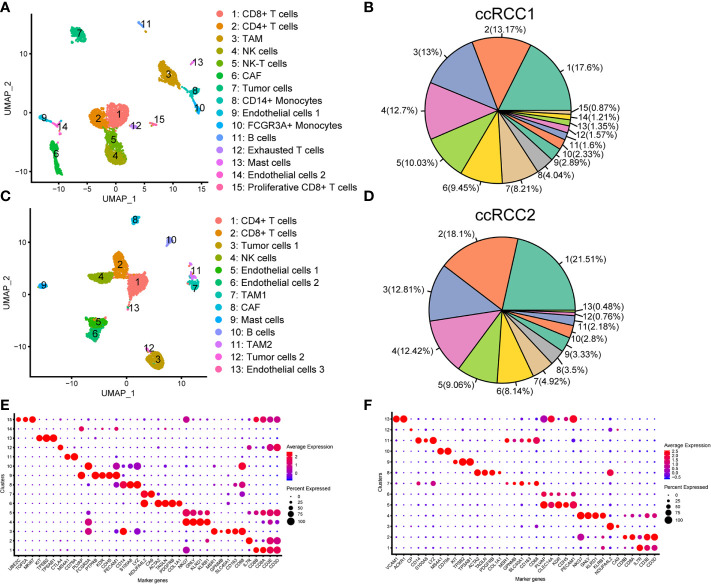
Single-cell transcriptomic map of sporadic bilateral ccRCC. **(A)** Uniform manifold approximation and projection (UMAP) plot representation of right tumour (ccRCC1) with 15 distinct cell types. **(B)** Proportion of each cell type in ccRCC1. **(C)** UMAP plot representation of left tumour (ccRCC2) with 13 distinct cell types. **(D)** Proportion of each cell type in ccRCC2. **(E, F)** Bubble chart showing the marker genes of each cluster in ccRCC1 **(E)** and ccRCC2 **(F)**; the selected marker genes for each cluster are highlighted.

scRNA-seq was conducted for the right (ccRCC1) and left (ccRCC2) renal tumour tissues by using the 10× Genomics technique (Methods). Sample ccRCC1 could capture 6,348 single-cells, and sample ccRCC2 could capture 7,775 single-cells. A total of 3,568 and 3,575 high quality single-cells were obtained in ccRCC1 and ccRCC2, respectively, after QC by Seurat (21, 22) (**Table 2**). Unbiased clustering classified the cells in ccRCC1 into 15 different types from cluster 1 to cluster 15, namely, CD8^+^ T cells, CD4^+^ T cells, tumour associated macrophages (TAMs), NK cells, NK-T cells, cancer-associated fibroblast (CAF), tumour cells, CD14+ monocytes, endothelial cells 1, FCGR3A+ monocytes, B cells, exhausted T cells, mast cells, endothelial cells 2 and proliferative CD8^+^ T cells (**Figure 3A**). The immune cells were abundant in ccRCC1, especially T cells, and could be classified into four subtypes (**Figures 3A, B**).

The cells in ccRCC2 can be classified into 13 different types, namely, CD4^+^ T cells, CD8^+^ T cells, tumour cells 1, NK cells, endothelial cells 1, endothelial cells 2, TAM1, CAF, mast cells, B cells, TAM2, tumour cells 2 and endothelial cells 3 (**Figure 3C**). Similar to that in ccRCC1, the number of immune cells was high (**Figure 3D**). Endothelial cells were classified into three different subtypes in CCRCC2 but only into two subtypes in ccRCC1. Two subtypes of tumour cells were found in ccRCC2 but only one subtype in ccRCC1. On the basis of its occurrence on genetic material in tumours at different anatomical sites (27), heterogeneity may alter the transcription of tumour cells. Thus, tumour cells can exhibit different gene expression characteristics, resulting in the generation of cell subtypes.

Cell type was defined by the expression of marker genes (15, 26) in the cell clusters. With the expression of marker genes and the proportion of cell expression as the basis, bubble diagram was drawn to display the characteristic genes of all cell types (**Figures 3E, F**).

### Tumour Cells Were Further Identified Through the CNV Analysis of scRNA-seq Results

**Figure 4:**
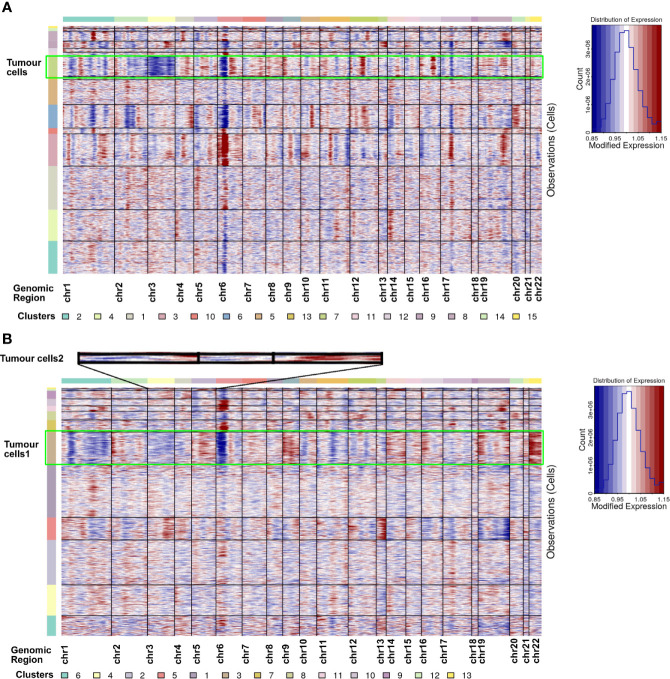
CNV analysis for all cell types in ccRCC1 **(A)** and ccRCC2 **(B)**, respectively. Copy number gains (red) and losses (blue).

Tumour cells were first identified according to the marker genes (*CA9* and *NDUFA4L2*) (28, 29). In ccRCC1, cluster 7 was identified as tumour cell type with highly expressed *CA9* and *NDUFA4L2* (**Figure 3E**). Two tumour cell subtypes, namely, cluster 3 and cluster 12 were discovered in ccRCC2 (**Figure 3F**). Tumour cells 2 had highly expressed *CA9* and *CP* but lowly expressed *NDUFA4L2* (**Figure 3F**). CNV analysis was conducted on the scRNA-seq results to further confirm the classification of tumour cells. The copy number of each cell type on autosomes was analysed. The findings showed that only tumour cells (cluster 7) involved the copy number losses of chromosome 3 in ccRCC1 (**Figure 4A**). In ccRCC2, chromosome 3 loss and chromosome 5 gain were highly remarkable in tumour cells 1 (cluster 3) and 2 (cluster 12) (**Figure 4B**). These occurrences are common copy number alterations of ccRCC (29). Thus, the classification of tumour cells was precise and reliable.

### Except for Tumour Cells, Other Cell Types Showed Extremely High Similarity in Both Types of Bilateral ccRCC

**Figure 5:**
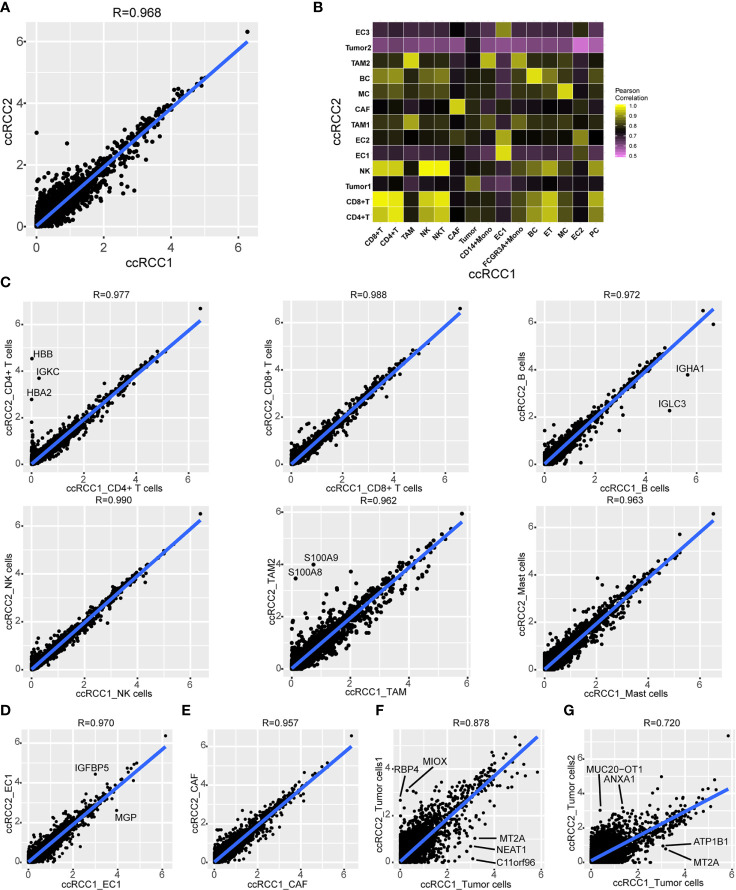
Comparison of gene expression in bilateral renal tumours. **(A)** Scatterplot showing the *log1p* of the average expression (AE) per gene in total ccRCC1 (horizontal) and ccRCC2 (vertical). Pearson’s correlation coefficient was 0.968 (R=0.968). **(B)** Heat map indicating Pearson correlations on the averaged profiles among each cell type for ccRCC1 (horizontal) and ccRCC2 (vertical). **(C, G)** Scatterplot showing the *log1p* of the average expression (AE) per gene of CD4^+^ T cells **(C)**, CD8^+^ T cells **(C)**, B cells **(C)**, NK cells **(C)**, TAM **(C)**, mast cells **(C)**, endothelial cells (EC**, D**), CAF **(E)** and tumour cells **(F, G)** in ccRCC1 (horizontal) and ccRCC2 (vertical).

The gene expression characteristics of tumour tissues on the left and right sides of bilateral ccRCC were investigated. Analysis on the mean gene expression of all cell types in bilateral ccRCC revealed high similarity between gene expression of left and right samples according to the Pearson’s correlation coefficient of 0.968 (**Figure 5A**).

Gene expression correlations were compared between each cell subtype (**Figure 5B**). A good correlation was observed between gene expression levels of the same cell type (**Figure 5B**). For example, high similarity was found between the immune cells of both bilateral tumours with Pearson’s correlation coefficients greater than 0.95 (**Figure 5C**). A high degree of similarity was also noted between CAF and endothelial cells from different tumour tissues with Pearson’s correlation coefficients of 0.970 and 0.957, respectively (**Figures 5D, E**). However, the similarity of gene expression between tumour cells was relatively low. The Pearson’s correlation coefficient between tumour cells in ccRCC1 and tumour cells 1 in ccRCC2 was 0.878 (**Figure 5F**), and that between tumour cells in ccRCC1 and tumour cells 2 in ccRCC2 was 0.720 (**Figure 5G**). Some genes, such as *MT2A*, *NEAT1*, *ATP1B1*, and *C11orf96*, were highly expressed in tumour cells from ccRCC1. On the contrary, *RBP4* and *MIOX* were highly expressed in tumour cells 1 from ccRCC2, and *ANXA1* and *MUC20-OT1* were highly expressed in tumour cells 2 from ccRCC2 but lowly expressed in tumour cells 2 from ccRCC1 (**Figures 5F, G**). Although the bulk gene expression in tumour tissues was highly correlated, the tumour cell types had significantly different gene expression within the tumour. Other identical cell types in both bilateral tumours, including immune cells, CAF and endothelial cells, had highly similar gene expression.

### scRNA-seq Revealed the Gene Expression Characteristics of Tumour Cells at Different Anatomical Sites in Bilateral ccRCC

**Figure 6:**
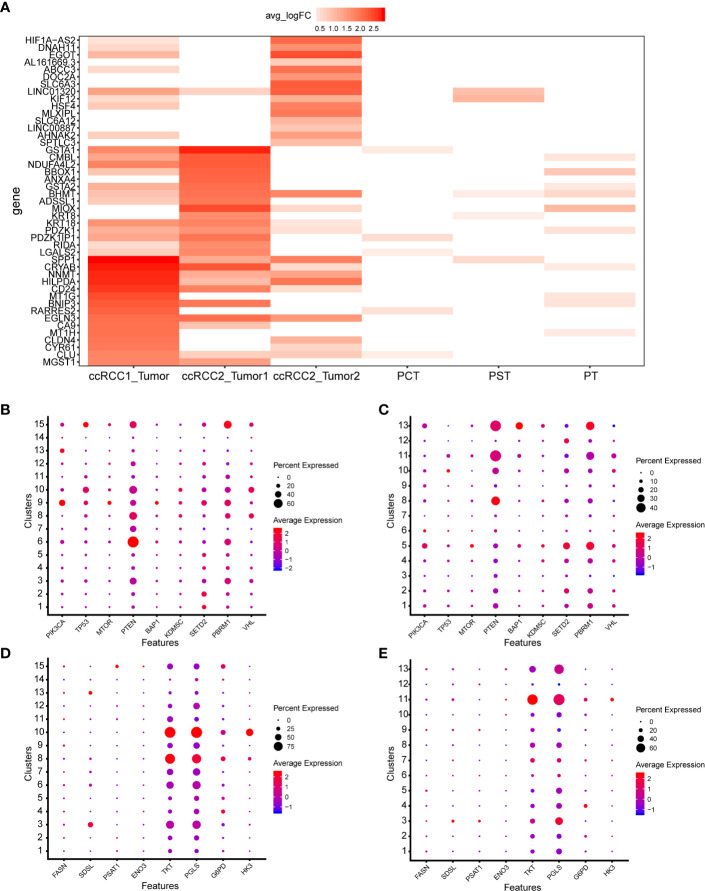
ScRNA-seq revealed the cellular molecular characteristics of bilateral ccRCC. **(A)** Comparison of DEGs in different types of tumour and normal kidney cells. Proximal convoluted tubule cells (PCT), proximal straight tubule cells (PST), proximal tubule cells (PT). **(B, C)** Expression of common mutated genes in ccRCC1 and ccRCC2. **(D, E)** Expression of metabolism related genes in ccRCC1 and ccRCC2.

scRNA-seq can precisely identify tumour cells and discover their specific gene expression. The genes with most significant differential expression in three tumour cell types were identified (**Figure 6A**). Tumour cells (ccRCC1) contained almost all of the genes that were highly expressed in the other two tumour cell types of ccRCC2; however, some of these genes were expressed at relatively low levels. Differences in gene expression between tumour cells 1 and 2 in ccRCC2 were highly evident (**Figure 6A**). *SPP1*, *CRYAB*, *NNMT* and *HILPDA* were highly expressed in tumour cells (ccRCC1); *GSTA2*, *BHMT* and *ADSSL1* were highly expressed in tumour cells 1 (ccRCC2); and *HIF1A-AS2*, *DNAH11* and *EGOT* were highly differentially expressed in tumour cells 2 (ccRCC2). *HIF1A-AS2* is an antisense RNA of factor hypoxia-inducible factor-1 alpha (HIF1A), which is closely related to the tumourigenesis and progression of ccRCC (22). This gene was highly expressed in tumour cells 2 but not in tumour cells 1 (**Figure 6A**), indicating intratumour heterogeneity. Interestingly, compared with normal kidney cells (15), DEGs of tumour cells were generally low or not expressed in normal kidney cells (**Figure 6A**).

Most common mutated genes in ccRCC had enriched expression at the single-cell level (29, 30) and had similar expression in bilateral ccRCC (**Figures 6B, C**). Most of the common mutated genes were lowly or even not expressed in all cell types (**Figures 6B, C**). Additionally, *PTEN* expression was cell-type specific and was high in the CAF of ccRCC1 and ccRCC2 (**Figures 6B, C**).

Given their association with prognosis, the expression of some metabolism-related genes in each type of cell of ccRCC1 and ccRCC2 was screened out (29). These genes were expressed similarly in bilateral ccRCC. For example, *TKT* and *PGLS* were highly expressed in monocyte–macrophage system whether in ccRCC1 or ccRCC2 (**Figures 6D, E**). HK3 was highly expressed in B cells (**Figures 6D, E**).

### GO Analysis of Bilateral Tumour Cells Was Conducted to Discover the Potential Biological Characteristics

**Figure 7:**
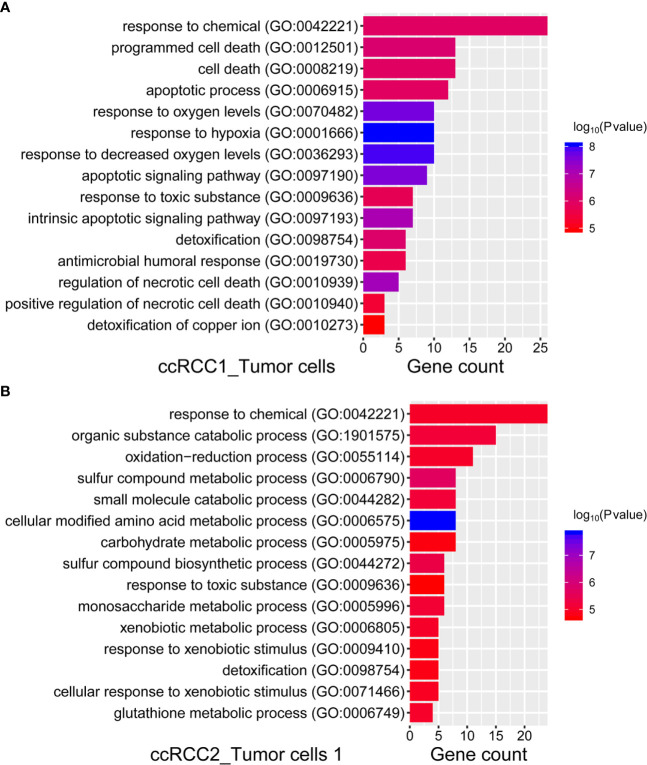
GO enrichment analysis of ccRCC1 and ccRCC2 tumour cells for biological process. **(A)** We presented the most significant 15 biological processes in ccRCC1 tumor cells, according to the top 50 DEGs, which were selected for GO enrichment analysis. **(B)** The most significant 15 biological processes in ccRCC2 tumor cells 1, according to the top 50 DEGs.

GO analysis (25) (http://geneontology.org/) was performed on different tumour cells on both sides to identify the biological process of tumour cells by DEGs. Given that tumour cells 2 in ccRCC2 failed in the enrichment of significant biological processes, only the most significant biological processes of tumour cells 2 in ccRCC1 and tumour cells 1 in ccRCC2 were presented (**Figure 7**). The enrichment of biological processes for tumour cells on both sides was the same, such as the ‘response to chemical,’ ‘response to toxic substance,’ and ‘detoxification’ (**Figure 7**). This finding indicated similar biological characteristics between the tumour cells on both sides. However, significant differences in biological processes were also noted. For example, ‘response to hypoxia’, ‘response to oxygen levels’ and ‘response to decreased oxygen levels’ were the most significant biological process in the tumour cells 2 of ccRCC1 (**Figure 7A**). This result suggested that the tumour cells 2 of ccRCC1 may be involved in hypoxia and was consistent with the mechanism of ccRCC in previous studies (31, 32). However, the tumour cells 1 of ccRCC2 enriched biological processes different from those of tumour cells 2 of ccRCC1. These biological characteristics were closely related to some metabolic processes, such as ‘cellular modified amino acid metabolic process’, ‘sulphur compound metabolic process’, ‘glutathione metabolic process’, ‘carbohydrate metabolic process’ and ‘monosaccharide metabolic process’ (**Figure 7B**).

## Discussion

A case of sporadic bilateral ccRCC was studied by single-cell transcriptome sequencing. Compared with many previous works on bilateral RCC (33–35) the present study firstly examined the tumour cellular biological characteristics of bilateral ccRCC at the single-cell level. Although large samples of DNA, bulk RNA and DNA methylation in ccRCC have been reported (29, 30) these results only revealed the characteristics of RCC from an average level; specifying these attributes to cell types, especially tumour cells, is difficult. With scRNA-seq, the complex intratumour cellular structure was identified, and the gene expression characteristics of tumour tissues in different sites were revealed. The expression of some key genes from cell types was explored through the unbiased clustering of cells. This method is more accurate and instructive than bulk RNA sequencing.

Different tumour cell types were found at various sites in the left and right tumour tissues. Only one tumour cell type was found in ccRCC1, and two tumour cell types were noted in ccRCC2 (**Figures 3A, C**). The tumour cells in varying parts may produce different gene expression levels during tumour evolution, thereby leading to the different biological functions of tumour cells that may reflect their microenvironment and disease prognosis. ScRNA-seq for tumours may be a method to address these problems. Although the WHO/ISUP grading of renal carcinoma has a substantial influence on patient prognosis (36), the correlation between the complexity of tumour cell types is unknown. This work found only one type of tumour cells in ccRCC1 with WHO/ISUP grade I and two types of tumour cells in ccRCC2 with WHO/ISUP grade II. Considering the limited number of samples, the relationship between WHO/ISUP grade and tumour cell types was not well explained.

In addition, it is a new breakthrough to apply single-cell sequencing technology to tumour research. Previously, researchers focused more on DNA variations in tumour tissue, which discovered many mutant genes and evolutionary subtypes (29, 37). However, how do these common mutated genes drive universal changes in the transcriptome and what specific genes are expressed in tumour cells? Obviously, the application of bulk RNA sequencing is difficult to accurately address these problems. Considering that tumour cells can be accurately identified by unbiased clustering, scRNA-seq can reveal the gene expression characteristics of tumour cells. The genes specifically high expressed in these tumour cells may be associated with the occurrence, development and progression of tumours. At the same time, the results of scRNA-seq can discover the potential biological function of tumour cells (**Figures 7A, B**). Thus, scRNA-seq of tumour tissue can provide clues to the diagnosis and treatment of tumours.

In summary, this study revealed the transcriptomic characteristics of tumour tissues in different locations at the single-cell transcriptome level by using the scRNA-seq data of bilateral sporadic ccRCC. The results provide new insights into the research of bilateral RCC.

## Data Availability Statement

The raw data and processed data in this article could be accessed in the NCBI GEO 'Datasets (GSE171306). The sequencing data also have been uploaded to the figshare database. It is possible to access these data through the website (https://doi.org/10.6084/m9.figshare.13649069). These data include barcodes.tsv, features.tsv and gene expression matrix (*.mtx) files.'

## Ethics Statement

The studies involving human participants were reviewed and approved by the institutional review board of The First Affiliated Hospital Guangxi Medical University. The patients/participants provided their written informed consent to participate in this study. Written informed consent was obtained from the individual(s) for the publication of any potentially identifiable images or data included in this article.

## Author Contributions

ZY performed scRNA-seq analyses, created the figures and wrote the paper. WL performed the scRNA-seq experiments and wrote the paper. CS and YL performed the scRNA-seq experiments. YY discussed the draft paper and critically reviewed the manuscript. BG provided assistance during establishing library. DL, HY, HM, TL and QZ provided and dissected RCC and human kidney tissues. JC and ZM conceived and supervised the project, analysed the data, created the figures and wrote the paper. All authors contributed to the article and approved the submitted version.

## Funding

This work was supported by the grants from the National Natural Science Foundation of China (81770759), the National Key R&D Program of China (2017YFC0908000), Major Project of Guangxi Innovation Driven (AA18118016), Guangxi key Laboratory for Genomic and Personalized Medicine [grant number 16-380-54, 17-259-45, 19-050-22, 19-185-33, 20-065-33], Guangxi Science and Technology Base and Talent Project (2019AC17009) and Guangxi Clinical Research Center for Urology and Nephrology (2020AC03006).

## Conflict of Interest

The authors declare that the research was conducted in the absence of any commercial or financial relationships that could be construed as a potential conflict of interest.
